# Decoding deception: the binding affinity of cuttlefish ink on shark smell receptors

**DOI:** 10.1093/g3journal/jkaf001

**Published:** 2025-01-08

**Authors:** Colleen Lawless, Lauren E Simonitis, John A Finarelli, Graham M Hughes

**Affiliations:** School of Biology and Environmental Science, University College Dublin, Belfield, Dublin 4, Ireland; Department of Biological Sciences, Florida Atlantic University, Boca Raton, FL 33431, USA; School of Biology and Environmental Science, University College Dublin, Belfield, Dublin 4, Ireland; School of Biology and Environmental Science, University College Dublin, Belfield, Dublin 4, Ireland

**Keywords:** ligand–receptor interaction, protein structure prediction, olfactory genes

## Abstract

Chemical signaling can play a crucial role in predator–prey dynamics. Here, we present evidence that ink from the common cuttlefish (*Sepia officinalis*) targets olfactory receptor proteins in sharks, potentially acting as a predator deterrent. We apply in silico 3D docking analysis to investigate the binding affinity of various odorant molecules to shark olfactory receptors of 2 shark species: cloudy catshark (*Scyliorhinus torazame*) and white shark (*Carcharodon carcharias*). Pavoninin-4 (a known shark repellent compound) displayed selectivity in binding to receptors in the white shark. In contrast, the primary component of cuttlefish ink, melanin, displayed the highest binding affinities to all shark olfactory receptor proteins in both species. Taurine, another important ink component, exhibited standard to strong bindings for both species. Trans-4,5-epoxy-(E)-2-decenal (“blood decenal”), an odorant associated with the smell of blood, displayed strong binding affinities to all shark olfactory receptors, similar to that of melanin. These findings provide new insights into the molecular interplay between cephalopod inking behavior and their shark predators, with cuttlefish ink likely exploiting the narrow band of the shark olfactory repertoire.

## Graphical Abstract

**Graphical Abstract:**
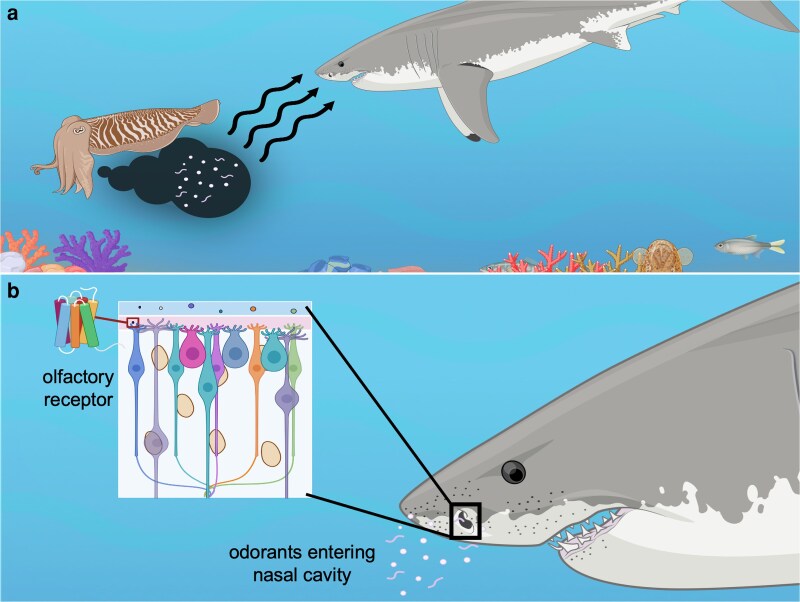

## Introduction

Chemical signals carry information about the environment, allowing animals to locate food, find mates, and avoid danger. As such, olfaction, the chemical sensory mode called “smell,” is essential for survival in both predator and prey species. For example, barnacles secrete glycoprotein compounds as a mechanical defense against predation, yet many of their predators (e.g. sea stars, whelks, and snails) have evolved olfactory receptors to detect these glycoproteins, allowing them to locate the barnacles more efficiently (Zimmer *et al*. 2021). However, reliance on olfaction can also present a vulnerability for predators. For example, the California sea hare, *Aplysia californica*, expels a dark liquid (“ink”) as a defensive strategy against spiny lobsters, which is thought to deter predation through a combination of phagomimicry and chemical sensory disruption (Derby 2007).

Olfactory receptors are chemosensory proteins belonging to the family of G-protein-coupled receptors and, in vertebrates, are often expressed in the cell membrane of the nasal epithelium (Firestein 2001). They play a vital role in processing chemical signals from the external environment. Activation, by binding with an agonist or antagonist molecule, initiates a signaling cascade that ultimately results in perception of a chemical stimulus (“smell”) (Peterlin *et al*. 2008). The 4 primary olfactory receptor gene families responsible for vertebrate olfaction are (1) odorant receptors (ORs), (2) trace amine-associated receptors (TAARs), (3) olfactory receptors related to class A (ORA, also: V1Rs), and (4) vomeronasal type 2 receptors (V2Rs, also: OlfCs). These receptor families were initially characterized in mammals (Buck and Axel 1991; Dulac and Axel 1995; Matsunami and Buck 1997; Liberles and Buck 2006) and have subsequently been recognized in other vertebrate groups, including birds (Hillier *et al*. 2004; Niimura and Nei 2005) and other tetrapod clades (Dehara *et al*. 2012), ray-finned fishes (Niimura 2009) and more recently sharks (Hara *et al*. 2018; Marra *et al*. 2019; Sharma *et al*. 2019; Syed *et al*. 2023).

Coleoid cephalopods (squid, octopus, and cuttlefish) are thought to have diverged from other mollusks ∼500 MYA (Derby 2014). While the loss of external shells has increased their mobility relative to other mollusk groups, it has also increased predation risk (Wells 1995). In response, coleoid cephalopods have evolved alternative predator deterrence mechanisms, notably ink (Derby *et al*. 2013). Ink released by the common cuttlefish (*Sepia officinalis*) is principally composed of melanin, the pigment responsible for its dark color, and multiple free amino acids, of which the nonproteinogenic amino acid taurine is found in the highest concentration (Derby 2007). Sharks (Chondrichthyes and Selachii) are significant cephalopod predators, and there is evidence showing that cuttlefish ink has an adverse effect on shark swimming behavior (Simonitis 2021).

Sharks have informally been referred to as “swimming noses.” The relatively large olfactory bulbs (OBs) observed in sharks have led to the popular notion of their having an acute sense of smell (Hodgson and Mathewson 1978). Recent sequencing of multiple shark genomes (Hara *et al*. 2018; Marra *et al*. 2019; Tan *et al*. 2021; Stanhope *et al*. 2023) has revealed a surprisingly low number of sensory-specific olfactory receptors compared with other vertebrate clades. Sharks exhibit an average of 43 total olfactory receptor genes, whereas ray-finned fishes typically average >200, and mammals ∼850 (Policarpo *et al*. 2023). As such, potential olfactory acuity and molecular breadth of total olfactory ability appear decoupled in this clade.

Here, we investigate the exploitation of predator olfactory receptors via ink in the shark–cuttlefish system. Using computational approaches, we modeled the 3D structures of olfactory receptor proteins from 2 ecologically distinct shark species, the cloudy catshark (*Scyliorhinus torazame*), and the white shark (*Carcharodon carcharias*). By examining monomolecular binding affinities for cuttlefish ink components, the known shark repellent compound pavoninin-4 (Tachibana *et al*. 1984), 2 odorants associated with putrefaction (cadaverine and putrescine) and the odorant responsible for the smell of blood (trans-4,5-epoxy-(E)-2-decenal, hereafter: “blood decenal”), we elucidate how a prey species may disrupt shark sensory perception through simultaneous binding across the entire suite of multiple divergent olfactory receptors. Our results highlight how 3D protein modeling can complement our understanding of the life history and ecology of predator–prey interactions.

## Materials and methods

### Gene selection, protein structure prediction, and odor docking

Olfactory receptor gene sequences (OR, TAAR, V1R/ORA, and V2R/OlfC) were selected from Sharma *et al*. (2019) and Syed *et al*. (2023), with receptor names assigned based on the small-spotted catshark (*Scyliorhinus canicula*) gene nomenclature. Genes were aligned using Clustal Omega (Sievers *et al*. 2014). Each alignment was used to generate phylogenetic trees using W-IQ-TREE, a server for maximum likelihood analysis using the IQ-TREE software, with 1,000 ultrafast nonparametric bootstrap replicates (Nguyen *et al*. 2014; Trifinopoulos *et al*. 2016) and the inferred best-fit model of sequence evolution. One representative receptor per subclade was then chosen across all olfactory gene families. This provided targets to examine odorant–receptor binding combinations using a variety of chemical compounds (volatile and nonvolatile odorants). Given that olfactory receptors are membrane-bound, it is difficult to isolate and resolve their atomic structure via X-ray crystallography. Therefore, binding combinations were investigated using in silico methods. 3D odorant structures used in this experiment were downloaded from PubChem (Kim *et al*. 2022; Supplementary Table 1). The following odorants were selected: (1) melanin secreted from common cuttlefish (*S. officinalis*) which gives ink the dark color, (2) taurine, a free nonproteinogenic amino acid found at millimolar concentrations in cuttlefish ink (Derby *et al*. 2013), (3) pavoninin-4, a toxin secreted from the Pacific sole (*Pardachirus pavoninus*; Williams *et al*. 2005), (4) cadaverine, (5) putrescine (Hussain *et al*. 2013), and (6) blood decenal (Sarrafchi and Laska 2016). Cadaverine was also used as a positive control to test the reliability of our odor docking assay, as it has previously been shown to have a high binding affinity for TAAR13c in zebrafish (*Danio rerio*; NP_001076509.1), the binding of which causes an avoidance response (Hussain *et al*. 2013). Based on this, the score obtained from initially docking cadaverine to the zebrafish TAAR13c 3D structure was used as the relative threshold for a high binding affinity.

Receptor 3D structures were predicted using their amino acid sequence (Supplementary material) as input to the Iterative Threading ASSEmbly Refinement (I-TASSER) protein modeling server (Yang and Zhang 2015; Zheng *et al*. 2021; Zhou *et al*. 2022). I-TASSER identifies structural templates from the PDB using multiple threading alignment approaches and attempts to infer the best possible structural prediction using homology modeling. Up to 5 structural models are predicted, each with an estimated global confidence score (*C*-score) that quantifies the reliability and quality of predicted models. Typically ranging between −5 and 2, a higher *C*-score indicates greater confidence in the model. We selected the model with the highest *C*-score for subsequent protein docking. Predicted ligand-binding sites were identified with COFACTOR (Roy *et al*. 2012; Zhang *et al*. 2017) and COACH (Yang *et al*. 2013) and implemented in I-TASSER. Custom cavity docking was performed by CB-dock2 (Liu *et al*. 2022), which predicts odorant docking contact sites with the protein. UCSF Chimera (Pettersen *et al*. 2004) was used for visualization and subsequent analyses. To investigate the olfactory potential of shark species, we performed custom molecular docking simulations for 6 odorant molecules: 3 environmental odorants potentially under selection (putrescine, cadaverine, and blood decenal); a known shark repellent odorant (pavoninin-4); and 2 molecules found in cuttlefish ink (taurine and melanin).

## Results

### Gene trees and selecting targets to model

A total of 146 olfactory receptor gene sequences were sourced from previously published data (Supplementary Table 2; Sharma *et al*. 2019; Syed *et al*. 2023). This dataset included multiple species, specifically the cloudy catshark, the white shark for subsequent protein modeling, and the small-spotted catshark, which served as a naming reference point for the receptors used in this study (Supplementary Fig. 1). The dataset included 29 ORs, 14 ORAs, 9 TAARs, and 94 V2Rs across all species. The mean amino acid residue length varied between the olfactory families: ORs have an average length of 303 amino acids, while the (multiexon) V2Rs are significantly longer at 643 amino acids. For subsequent protein modeling efforts, the final selection of receptors consisted of 1 OR, 1 TAAR, 1 ORA, and 4 V2Rs sourced from both shark species analyzed (Supplementary Fig. 1).

### In silico binding of the death-associated odor cadaverine to zebrafish TAAR13c

The death-associated odor compound cadaverine has been shown to bind strongly in vivo to the zebrafish TAAR13c receptor (Hussain *et al.* 2013). We modeled the 3D structure of zebrafish TAAR13c, and docked cadaverine, in silico, as a positive control for the reliability of our computational docking assay. COFACTOR (Roy *et al*. 2012; Zhang *et al*. 2017) and COACH (Yang *et al*. 2013) identified 38 contact residues within the upper third portion of the transmembrane domain of TAAR13c (Supplementary Table 3). The docking results highlighted Asp112 and Trp296 (located on transmembrane 7 and transmembrane 8, respectively) as the key residues for cadaverine binding, in agreement with previous studies that predicted these residues as binding partners (Huang 2003; Sharma *et al*. 2016; Supplementary Fig. 2). Docking cadaverine with zebrafish TAAR13c resulted in a docking score of −4.0 (where more negative scores represent greater binding affinities). We used this score as a threshold indicator of high ligand–receptor binding affinity. It is important to note 1 limitation of in silico docking experiments here. Geometric fit of ligands at the active site is measured under a static model (Terefe and Ghosh 2022); therefore, dockings generally do not account for protein flexibility, which in turn can affect the physiochemical environment in the binding region.

### In silico binding assay to shark ORs

**Fig. 1.**
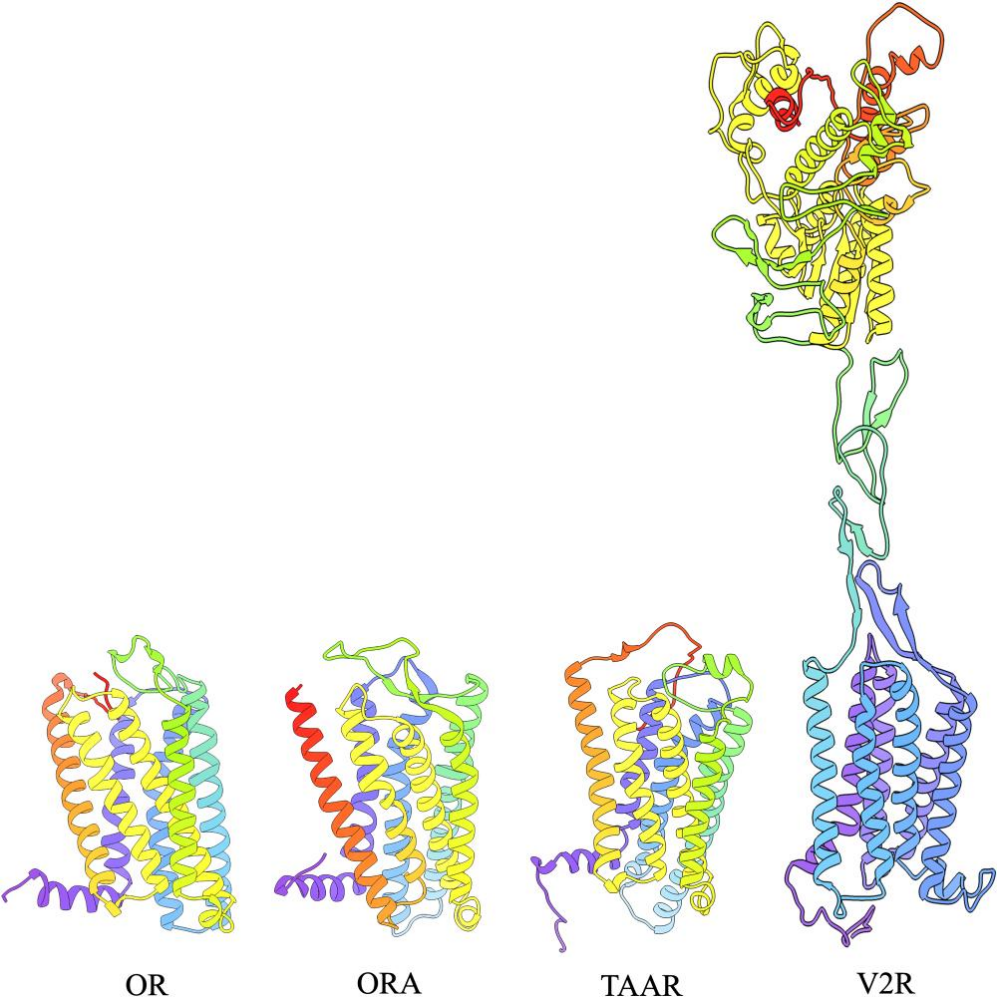
Representative predictive 3D receptor structures of 4 major olfactory receptor types found in the white shark OR, TAAR, and V2R genes are generally encoded by a single exon and consist of 7 transmembrane helices, whereas V2R genes are generally encoded by 6–7 exons and possess long N-terminal tails. OR, odorant receptor; TAAR, trace amine-associated receptor; ORA, olfactory receptors related to class A; V2R, vomeronasal type 2 receptor.

**Fig. 2.**
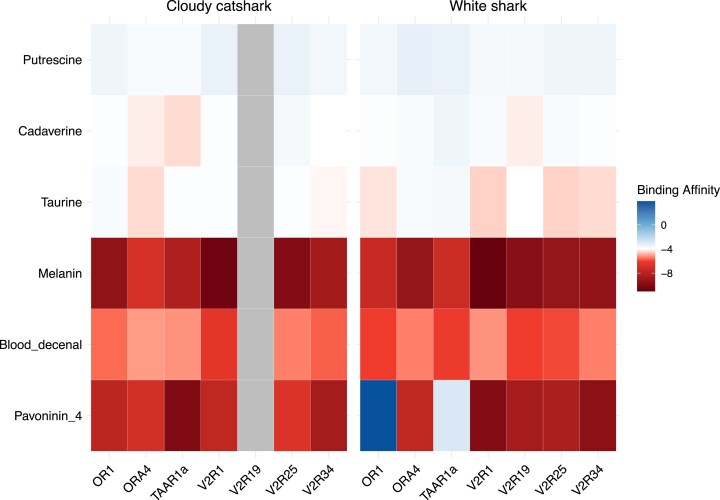
Odorant–receptor heat map. Each shark receptor was docked with 6 different odorants (putrescine, cadaverine, taurine, melanin, blood decenal, and pavoninin-4) to examine the binding affinity. Each row represents an odorant, and each column represents an olfactory receptor. The zebrafish/cadaverine complex was used to indicate successful binding (−4 shown in white). High binding affinity is represented by warmer tones, lower binding affinity by cooler tones and non-functional genes are indicated by a tone that a distinct from the gradient extremes.

Protein structure predictions for each shark olfactory receptor were generated with I-TASSER (Fig. 1; Yang and Zhang 2015; Zheng *et al*. 2021; Zhou *et al*. 2022). The reliability of each model was assessed using the *C*-score, with higher scores indicating greater confidence (Supplementary Table 4). Predicted ligand-binding sites for shark olfactory receptors are given in Supplementary Table 5. There were minute differences in binding patterns between the 2 species (Supplementary Table 6 and Fig. 2). Odorant docking scores correlate with binding free energy; therefore, more negative scores indicate stronger binding of the odorant to the olfactory receptor protein (García-Ortegón *et al*. 2022). Melanin and blood decenal showed the highest binding affinities, with docking scores ≤−4.0, across all receptors for both shark species (Fig. 2). Taurine and cadaverine exhibited moderate-to-high binding affinities for different receptor–odorant combinations. Putrescine displayed moderate binding affinities across all receptors in both shark species. One discrepancy in the pattern of binding between sharks was observed for pavoninin-4. The white shark displayed very low binding affinity (docking score: 3.9) for the OR1 receptor, whereas this OR1 showed high affinity (−7.8) in the cloudy catshark (Supplementary Table 6).

## Discussion

Sharks exhibit a range of relative OB sizes, depending on ecology and habitat (Yopak *et al*. 2010, 2014), with the white shark, a large-bodied, high-speed predator in coastal/oceanic habitats, possessing one of the largest relative OB sizes among sharks (Yopak *et al*. 2014). Among vertebrates, a larger relative size for sensory processing regions of the brain (e.g. OB and optic tectum) is thought to reflect an increased reliance on that particular sensory mode (Cobb 1960) and increased relative OB size is often assumed to be evidence for increased olfactory acuity (Hodgson and Mathewson 1978). Similarly, there is an assumption on the genomic side that olfactory acuity is related to overall olfactory receptor gene count (Trimmer *et al*. 2019); that is, acuity and the potential breadth of molecules that can be detected are coupled. There is some evidence for a correlation between OB size and total OR count for some vertebrae groups, notably birds and mammals (Hughes *et al*. 2018; Hughes and Finarelli 2019). Yet sensory gene mining in recently sequenced shark genomes (Hara *et al*. 2018; Marra *et al*. 2019; Sharma *et al*. 2019; Syed *et al*. 2023) has demonstrated that sharks do not adhere to this pattern, with large olfactory architecture, acute sense of smell, and breadth of the deployed protein repertoire not being disassociated in this clade. One possible hypothesis is that sharks employ a very restricted set of finely tuned receptors, in lieu of the highly expanded repertoires observed in other vertebrate groups (e.g. mammals; Hughes *et al*. 2018). It should also be noted that neuron density in the olfactory structures of sharks exceeds that in mammals, which suggests a highly specialized olfactory system (Aicardi *et al*. 2024).

Understanding how different odor molecules interact with a broad range of olfactory receptors to trigger specific responses can inform our understanding of predator–prey interactions. Pavoninin-4 is an ichthyotoxin secreted by the peacock sole (*P. pavoninus*) to repel predators and was researched in the wake of World War II as a potential shark repellant (Tachibana *et al*. 1984). Pavoninin-4 has been shown to cause a state of tonic immobility when introduced into the nares of the lemon shark (*Negaprion brevirostris*; Tachibana *et al*. 1984, 1985; Tachibana and Gruber 1988; Hart and Collin 2015). Therefore, the potential exists for prey species to employ molecules to adversely affect the sensory systems of predator species as a deterrent. 3D modeling of shark olfactory receptor proteins allows us to estimate binding affinities with potential odorant components in cuttlefish ink. Unsurprisingly, pavoninin-4 has strong binding affinities across most of the shark olfactory receptor proteins, with only OR1 and TAAR1a in the white shark showing weak binding (Fig. 2). Both melanin and taurine bind across the entire repertoire of olfactory receptor proteins, with melanin displaying very high binding scores uniformly across both species (Fig. 2). This is despite the fact that the cloudy catshark and the white shark are ecologically distinct (Park *et al*. 2019; Guttridge *et al*. 2024), and their lineages diverged ∼150 MYA (Marjanović 2021). This agrees with previous research showing generally low genetic mutation rates in chondrichthyans (Martin *et al*. 1992; Hara *et al*. 2018; Sendell-Price *et al*. 2023). This is particularly intriguing, as the limited receptor repertoire in sharks should present fewer potential binding pocket configurations. Therefore, there is a limited, but also highly conserved, space of physiochemical characteristics presented by shark ORs across species.

The uniformly high binding affinities observed for blood decenal demonstrate its potential importance as an agonist in the shark olfactory system and also reveal the potential vulnerability to hack the entire (narrow) range of shark olfactory proteins with a single molecule. Cuttlefish have likely exploited this with melanin in their ink. Cuttlefish ink has been shown to induce avoidant swimming responses in sharks (Simonitis 2021). Melanin exhibits the same pattern of docking with all available receptor proteins as blood decenal, but it does so with stronger computed affinities. Receptor binding to OR proteins is not a binary “on/off” switch, but rather a complex, nonlinear process, with stronger binding affinities hypothesized to cause prolonged and more intense activations (Buchwald 2019; Du *et al*. 2023; Alsedfy *et al*. 2024). This suggests that melanin may induce a comparably stronger olfactory sensation than blood in sharks, that this sensation is aversive, and that it effectively obstructs the entire olfactory apparatus. Alternatively, melanin rich in amino acids may act as a phagomimic (Derby 2007; Wood *et al*. 2010). By activating similar olfactory receptors and signaling pathways as prey molecules, melanin could trigger feeding-associated responses in the shark, potentially misdirecting its attention toward the ink cloud and away from the inking animal. However, given the observed aversion response of sharks to cephalopod ink (Simonitis 2021), it is more likely that melanin primarily functions as a sensory disruptor.

The substantially lower counts of olfactory receptor genes in shark genomes, compared with other vertebrate groups, combined with slow rates of genomic evolution (Martin *et al*. 1992; Hara *et al*. 2018; Sendell-Price *et al*. 2023), suggest that olfactory capabilities are effectively fixed in this group and are not influenced by ecological factors. Cuttlefish ink has evolved to exploit a restricted and slowly evolving set of genes that presents an effectively stationary target in an evolutionary context. Our docking experiment results imply that melanin is the likely molecular mechanism of action. The demonstrated potential for ink to overcrowd the shark's olfactory perception, leading to repulsion and avoidance, represents a key element in the evolution of shark–cephalopod predatory interaction, highlighting the importance of computational biology and protein modeling in understanding prey defense at a molecular level.

## Supplementary Material

jkaf001_Supplementary_Data

## Data Availability

All supplementary files are available as part of the Supplementary material in this document. PDB files for 3D protein structures are available in Figshare, along with the alignment files at: https://doi.org/10.6084/m9.figshare.27934341.v1. Supplemental material available at G3 online.

## References

[jkaf001-B1] Aicardi S, Bozzo M, Guallart J, Garibaldi F, Lanteri L, Terzibasi E, Bagnoli S, Dionigi F, Steffensen JF, Poulsen AB, et al 2024. The olfactory system of sharks and rays in numbers. Anat Rec. 10.1002/ar.25537. 39030913

[jkaf001-B2] Alsedfy MY, Ebnalwaled AA, Moustafa M, Said AH. 2024. Investigating the binding affinity, molecular dynamics, and ADMET properties of curcumin-IONPs as a mucoadhesive bioavailable oral treatment for iron deficiency anemia. Sci Rep. 14(1):22027. 10.1038/s41598-024-72577-8. 39322646 PMC11424638

[jkaf001-B3] Buchwald P . 2019. A receptor model with binding affinity, activation efficacy, and signal amplification parameters for complex fractional response versus occupancy data. Front Pharmacol. 10:605. 10.3389/fphar.2019.00605. 31244653 PMC6580154

[jkaf001-B4] Buck L, Axel R. 1991. A novel multigene family may encode odorant receptors: a molecular basis for odor recognition. Cell. 65(1):175–187. 10.1016/0092-8674(91)90418-x. 1840504

[jkaf001-B5] Cobb S . 1960. Observations on the comparative anatomy of the avian brain. Perspect Biol Med. 3(3):383–408. 10.1353/pbm.1960.0053. 13810776

[jkaf001-B6] Dehara Y, Hashiguchi Y, Matsubara K, Yanai T, Kubo M, Kumazawa Y. 2012. Characterization of squamate olfactory receptor genes and their transcripts by the high-throughput sequencing approach. Genome Biol Evol. 4(4):602–616. 10.1093/gbe/evs041. 22511035 PMC3342882

[jkaf001-B7] Derby CD . 2007. Escape by inking and secreting: marine molluscs avoid predators through a rich array of chemicals and mechanisms. Biol Bull. 213(3):274–289. 10.2307/25066645. 18083967

[jkaf001-B8] Derby CD . 2014. Cephalopod ink: production, chemistry, functions and applications. Mar Drugs. 12(5):2700–2730. 10.3390/md12052700. 24824020 PMC4052311

[jkaf001-B9] Derby CD, Tottempudi M, Love-Chezem T, Wolfe LS. 2013. Ink from longfin inshore squid, *Doryteuthis pealeii*, as a chemical and visual defense against two predatory fishes, summer flounder, *Paralichthys dentatus*, and sea catfish, Ariopsis felis. Biol Bull. 225(3):152–160. 10.1086/bblv225n3p152. 24445441

[jkaf001-B10] Du R, Li L, Ji J, Fan Y. 2023. Receptor–ligand binding: effect of mechanical factors. Int J Mol Sci. 24(10):9062–9062. 10.3390/ijms24109062. 37240408 PMC10219515

[jkaf001-B11] Dulac C, Axel R. 1995. A novel family of genes encoding putative pheromone receptors in mammals. Cell. 83(2):195–206. 10.1016/0092-8674(95)90161-2. 7585937

[jkaf001-B12] Firestein S . 2001. How the olfactory system makes sense of scents. Nature. 413(6852):211–218. 10.1038/35093026. 11557990

[jkaf001-B13] García-Ortegón M, Simm GNC, Tripp AJ, Hernández-Lobato JM, Bender A, Bacallado S. 2022. DOCKSTRING: easy molecular docking yields better benchmarks for ligand design. J Chem Inf Model. 62(15):3486–3502. 10.1021/acs.jcim.1c01334. 35849793 PMC9364321

[jkaf001-B14] Guttridge TL, Matich P, Guttridge AE, Winton M, Dedman S, Skomal G. 2024. First evidence of white sharks, *Carcharodon carcharias*, in the tongue of the ocean, central Bahamas. Front Mar Sci. 11:1451808. 10.3389/fmars.2024.1451808.

[jkaf001-B15] Hara Y, Yamaguchi K, Onimaru K, Kadota M, Koyanagi M, Keeley SD, Tatsumi K, Tanaka K, Motone F, Kageyama Y, et al 2018. Shark genomes provide insights into elasmobranch evolution and the origin of vertebrates. Nat Ecol Evol. 2(11):1761–1771. 10.1038/s41559-018-0673-5. 30297745

[jkaf001-B16] Hart NS, Collin SP. 2015. Sharks senses and shark repellents. Integr Zool. 10(1):38–64. 10.1111/1749-4877.12095. 24919643

[jkaf001-B17] Hillier LW, Miller W, Birney E; International Chicken Genome Sequencing Consortium. 2004. Sequence and comparative analysis of the chicken genome provide unique perspectives on vertebrate evolution. Nature. 432(7018):695–716. 10.1038/nature03154. 15592404

[jkaf001-B18] Hodgson ES, Mathewson RF. 1978. Sensory Biology of Sharks, Skates, and Rays. Arlington (VA): U.S. Office of Naval Research.

[jkaf001-B19] Huang ES . 2003. Construction of a sequence motif characteristic of aminergic G protein-coupled receptors. Protein Sci. 12(7):1360–1367. 10.1110/ps.0305603. 12824482 PMC2323918

[jkaf001-B20] Hughes GM, Boston ESM, Finarelli JA, Murphy WJ, Higgins DG, Teeling EC. 2018. The birth and death of olfactory receptor gene families in mammalian niche adaptation. Mol Biol Evol. 35(6):1390–1406. 10.1093/molbev/msy028. 29562344 PMC5967467

[jkaf001-B21] Hughes GM, Finarelli JA. 2019. Olfactory receptor repertoire size in dinosaurs. Proc R Soc Lond B Biol Sci. 286(1904):20190909. 10.1098/rspb.2019.0909. PMC6571463 31185870

[jkaf001-B22] Hussain A, Saraiva LR, Ferrero DM, Ahuja G, Krishna VS, Liberles SD, Korsching SI. 2013. High-affinity olfactory receptor for the death-associated odor cadaverine. Proc Natl Acad Sci U S A. 110(48):19579–19584. 10.1073/pnas.1318596110. 24218586 PMC3845148

[jkaf001-B23] Kim S, Chen J, Cheng T, Gindulyte A, He J, He S, Li Q, Shoemaker BA, Thiessen PA, Yu B, et al 2022. PubChem 2023 update. Nucleic Acids Res. 51(D1):D1373–D1380. 10.1093/nar/gkac956. PMC9825602 36305812

[jkaf001-B24] Liberles SD, Buck LB. 2006. A second class of chemosensory receptors in the olfactory epithelium. Nature. 442(7103):645–650. 10.1038/nature05066. 16878137

[jkaf001-B25] Liu Y, Yang X, Gan J, Chen S, Xiao Z-X, Cao Y. 2022. CB-Dock2: improved protein–ligand blind docking by integrating cavity detection, docking and homologous template fitting. Nucleic Acids Res. 50(W1):W159–W164. 10.1093/nar/gkac394. 35609983 PMC9252749

[jkaf001-B26] Marjanović D . 2021. The making of calibration sausage exemplified by recalibrating the transcriptomic timetree of jawed vertebrates. Front Genet. 12:521693. 10.3389/fgene.2021.521693. 34054911 PMC8149952

[jkaf001-B27] Marra NJ, Stanhope MJ, Jue NK, Wang M, Sun Q, Pavinski Bitar P, Richards VP, Komissarov A, Rayko M, Kliver S, et al 2019. White shark genome reveals ancient elasmobranch adaptations associated with wound healing and the maintenance of genome stability. Proc Natl Acad Sci U S A. 116(10):4446–4455. 10.1073/pnas.1819778116. 30782839 PMC6410855

[jkaf001-B28] Martin AP, Naylor GJP, Palumbi SR. 1992. Rates of mitochondrial DNA evolution in sharks are slow compared with mammals. Nature. 357(6374):153–155. 10.1038/357153a0. 1579163

[jkaf001-B29] Matsunami H, Buck LB. 1997. A multigene family encoding a diverse array of putative pheromone receptors in mammals. Cell. 90(4):775–784. 10.1016/s0092-8674(00)80537-1. 9288756

[jkaf001-B30] Nguyen L-T, Schmidt HA, von Haeseler A, Minh BQ. 2014. IQ-TREE: a fast and effective stochastic algorithm for estimating maximum-likelihood phylogenies. Mol Biol Evol. 32(1):268–274. 10.1093/molbev/msu300. 25371430 PMC4271533

[jkaf001-B31] Niimura Y . 2009. On the origin and evolution of vertebrate olfactory receptor genes: comparative genome analysis among 23 chordate species. Genome Biol Evol. 1:34–44. 10.1093/gbe/evp003. 20333175 PMC2817399

[jkaf001-B32] Niimura Y, Nei M. 2005. Evolutionary dynamics of olfactory receptor genes in fishes and tetrapods. Proc Natl Acad Sci U S A. 102(17):6039–6044. 10.1073/pnas.0501922102. 15824306 PMC1087945

[jkaf001-B33] Park JM, Baeck GW, Raoult V. 2019. First observation on the diet and feeding strategy of cloudy catshark *Scyliorhinus torazame* (Tanaka, 1908). Reg Stud Mar Sci. 28:100596. 10.1016/j.rsma.2019.100596.

[jkaf001-B34] Peterlin Z, Li Y, Sun G, Shah R, Firestein S, Ryan K. 2008. The importance of odorant conformation to the binding and activation of a representative olfactory receptor. Chem Biol. 15(12):1317–1327. 10.1016/j.chembiol.2008.10.014. 19101476 PMC2628580

[jkaf001-B35] Pettersen EF, Goddard TD, Huang CC, Couch GS, Greenblatt DM, Meng EC, Ferrin TE. 2004. UCSF Chimera—a visualization system for exploratory research and analysis. J Comput Chem. 25(13):1605–1612. 10.1002/jcc.20084. 15264254

[jkaf001-B36] Policarpo M, Baldwin MW, Casañe D, Salzburger W. 2023. Diversity and evolution of the vertebrate chemoreceptor gene repertoire. Nat Commun. 15:1421. 10.21203/rs.3.rs-2922188/v1. PMC10869828 38360851

[jkaf001-B37] Roy A, Yang J, Zhang Y. 2012. COFACTOR: an accurate comparative algorithm for structure-based protein function annotation. Nucleic Acids Res. 40(W1):W471–W477. 10.1093/nar/gks372. 22570420 PMC3394312

[jkaf001-B38] Sarrafchi A, Laska M. 2016. Olfactory sensitivity for the mammalian blood odor component trans-4,5-epoxy-(E)-2-decenal in CD-1 mice. Perception. 46(3–4):333–342. 10.1177/0301006616653136. 27251166

[jkaf001-B39] Sendell-Price AT, Tulenko FJ, Pettersson M, Kang D, Montandon M, Winkler S, Kulb K, Naylor GP, Phillippy A, Fedrigo O, et al 2023. Low mutation rate in epaulette sharks is consistent with a slow rate of evolution in sharks. Nat Commun. 14(1):6628. 10.1038/s41467-023-42238-x. 37857613 PMC10587355

[jkaf001-B40] Sharma K, Ahuja G, Hussain A, Balfanz S, Baumann A, Korsching SI. 2016. Elimination of a ligand gating site generates a supersensitive olfactory receptor. Sci Rep. 6(1):28359. 10.1038/srep28359. 27323929 PMC4914996

[jkaf001-B41] Sharma K, Syed AS, Ferrando S, Mazan S, Korsching SI. 2019. The chemosensory receptor repertoire of a true shark is dominated by a single olfactory receptor family. Genome Biol Evol. 11(2):398–405. 10.1093/gbe/evz002. 30649300 PMC6368271

[jkaf001-B42] Sievers F, Wilm A, Dineen D, Gibson TJ, Karplus K, Li W, Lopez R, McWilliam H, Remmert M, Soding J, et al 2014. Fast, scalable generation of high-quality protein multiple sequence alignments using Clustal Omega. Mol Syst Biol. 7(1):539–539. 10.1038/msb.2011.75. PMC3261699 21988835

[jkaf001-B43] Simonitis LE . 2021. The neurobehavioral mechanisms of ink as an antipredation [Thesis]. Graduate and Professional School of Texas A&M University.

[jkaf001-B44] Stanhope MJ, Ceres KM, Sun Q, Wang M, Zehr JD, Marra NJ, Wilder AP, Zou C, Bernard AM, Pavinski-Bitar P, et al 2023. Genomes of endangered great hammerhead and shortfin mako sharks reveal historic population declines and high levels of inbreeding in great hammerhead. iScience. 26(1):105815. 10.1016/j.isci.2022.105815. 36632067 PMC9826928

[jkaf001-B45] Syed AS, Sharma K, Policarpo M, Ferrando S, Casane D, Korsching SI. 2023. Ancient and nonuniform loss of olfactory receptor expression renders the shark nose a de facto vomeronasal organ. Mol Biol Evol. 40(4):msad076. 10.1093/molbev/msad076. 36971115 PMC10116579

[jkaf001-B46] Tachibana K, Gruber SH. 1988. Shark repellent lipophilic constituents in the defense secretion of the Moses sole (*Pardachirus marmoratus*). Toxicon. 26(9):839–853. 10.1016/0041-0101(88)90325-x. 3201487

[jkaf001-B47] Tachibana K, Sakaitanai M, Nakanishi K. 1984. Pavoninins: shark-repelling ichthyotoxins from the defense secretion of the Pacific sole. Science. 226(4675):703–705. 10.1126/science.226.4675.703. 17774948

[jkaf001-B48] Tachibana K, Sakaitani M, Nakanishi K. 1985. Pavoninins, shark-repelling and ichthyotoxic steroid n-acetylglucosaminides from the defense secretion of the sole *Pardachirus pavoninus* (soleidae). Tetrahedron. 41(6):1027–1037. 10.1016/s0040-4020(01)96470-1.

[jkaf001-B49] Tan M, Redmond AK, Dooley H, Nozu R, Sato K, Kuraku S, Koren S, Phillippy AM, Dove AD, Read T. 2021. The whale shark genome reveals patterns of vertebrate gene family evolution. eLife. 10:e65394. 10.7554/elife.65394. 34409936 PMC8455134

[jkaf001-B50] Terefe EM, Ghosh A. 2022. Molecular docking, validation, dynamics simulations, and pharmacokinetic prediction of phytochemicals isolated from *Croton dichogamus* against the HIV-1 reverse transcriptase. Bioinform Biol Insights. 16:1779322221125605. 10.1177/11779322221125605. PMC9516429 36185760

[jkaf001-B51] Trifinopoulos J, Nguyen L-T, von Haeseler A, Minh BQ. 2016. W-IQ-TREE: a fast online phylogenetic tool for maximum likelihood analysis. Nucleic Acids Res. 44(W1):W232–W235. 10.1093/nar/gkw256. 27084950 PMC4987875

[jkaf001-B52] Trimmer C, Keller A, Murphy NR, Snyder LL, Willer JR, Nagai MH, Katsanis N, Vosshall LB, Matsunami H, Mainland JD. 2019. Genetic variation across the human olfactory receptor repertoire alters odor perception. Proc Natl Acad Sci U S A. 116(19):201804106. 10.1073/pnas.1804106115. PMC6511007 31040214

[jkaf001-B53] Wells MJ . 1995. The evolution of a racing snail. Mar Freshw Behav Physiol. 25(1–3):1–12. 10.1080/10236249409378904.

[jkaf001-B54] Williams JR, Gong H, Hoff N, Olubodun OI. 2005. Synthesis of the shark repellent pavoninin-4. J Org Chem. 70(26):10732–10736. 10.1021/jo051733a. 16355993

[jkaf001-B55] Wood JB, Maynard AE, Lawlor AG, Sawyer EK, Simmons DM, Pennoyer KE, Derby CD. 2010. Caribbean reef squid, *Sepioteuthis sepioidea*, use ink as a defense against predatory French grunts, *Haemulon flavolineatum*. J Exp Marine Biol Ecol. 388(1–2):20–27. 10.1016/j.jembe.2010.03.010.

[jkaf001-B56] Yang J, Roy A, Zhang Y. 2013. Protein–ligand binding site recognition using complementary binding-specific substructure comparison and sequence profile alignment. Bioinformatics. 29(20):2588–2595. 10.1093/bioinformatics/btt447. 23975762 PMC3789548

[jkaf001-B57] Yang J, Zhang Y. 2015. I-TASSER server: new development for protein structure and function predictions. Nucleic Acids Res. 43(W1):W174–W181. 10.1093/nar/gkv342. 25883148 PMC4489253

[jkaf001-B58] Yopak KE, Lisney TJ, Collin SP. 2014. Not all sharks are “swimming noses”: variation in olfactory bulb size in cartilaginous fishes. Brain Struct Funct. 220(2):1127–1143. 10.1007/s00429-014-0705-0. 24435575

[jkaf001-B59] Yopak KE, Lisney TJ, Darlington RB, Collin SP, Montgomery JC, Finlay BL. 2010. A conserved pattern of brain scaling from sharks to primates. Proc Natl Acad Sci U S A. 107(29):12946–12951. 10.1073/pnas.1002195107. 20616012 PMC2919912

[jkaf001-B60] Zhang C, Freddolino PL, Zhang Y. 2017. COFACTOR: improved protein function prediction by combining structure, sequence and protein–protein interaction information. Nucleic Acids Res. 45(W1):W291–W299. 10.1093/nar/gkx366. 28472402 PMC5793808

[jkaf001-B61] Zheng W, Zhang C, Li Y, Pearce R, Bell EW, Zhang Y. 2021. Folding non-homologous proteins by coupling deep-learning contact maps with I-TASSER assembly simulations. Cell Rep Methods. 1(3):100014. 10.1016/j.crmeth.2021.100014. 34355210 PMC8336924

[jkaf001-B62] Zhou X, Zheng W, Li Y, Pearce R, Zhang C, Bell EW, Zhang G, Zhang Y. 2022. I-TASSER-MTD: a deep-learning-based platform for multi-domain protein structure and function prediction. Nat Protoc. 17(10):2326–2353. 10.1038/s41596-022-00728-0. 35931779

[jkaf001-B63] Zimmer RK, Ferrier GA, Zimmer CA. 2021. Chemosensory exploitation and predator-prey arms races. Front Ecol Evol. 9:752327. 10.3389/fevo.2021.752327.

