# Supplementary material for: Decoding deception: the binding affinity of cuttlefish ink on shark smell receptors
Source: G3 (Bethesda). 2025 Jan 8;15(3):jkaf001. doi: 10.1093/g3journal/jkaf001 (PMC11917480; doi:10.1093/g3journal/jkaf001)
Supplement: jkaf001_Supplementary_Data [file jkaf001_supplementary_data.docx]

# Supplementary Material for Decoding Deception: The binding affinity of cuttlefish ink on shark smell receptors

**Supplementary Tables:**

| **PubChem ID** | **Synonyms** | **Chemical composition** |
| --- | --- | --- |
| 273 | Cadaverine | C_5_H_14_N_2_ |
| 6325610 | Melanin (*Sepia officinalis*) | C_18_H_10_N_2_O_4_ |
| 11578357 | Pavoninin-4 | C_37_H_63_NO_9_ |
| 1045 | Putrescine | C_4_H_12_N_2_ |
| 1123 | Taurine | C_2_H_7_NO_3_S |
| 526708 | Trans-4,5-epoxy-2(E)-decenal | C_10_H_16_O_2_ |

**Table S1. Chemical compounds used in the molecular docking assay.** Each chemical compound was downloaded from PubChem (https://pubchem.ncbi.nlm.nih.gov) and docked with 3D modelled chemosensory receptors.

| **Species** | **ORs** | **ORAs** | **TAARs** | **V2Rs** |
| --- | --- | --- | --- | --- |
| Cloudy catshark | 10 | 4 | 3 | 27 |
| White shark | 11 | 4 | 3 | 28 |
| Small-spotted catshark | 8 | 6 | 3 | 39 |

**Table S2. Total number of olfactory receptor gene sequences.** Sequences were taken from previously published work (Sharma et al. 2019; Syed et al. 2023), with the small-spotted catshark used as a naming reference for the gene trees in figure S1.

| **Odorant/receptor combination** | **Contact sites** |
| --- | --- |
| Cadaverina-TAAR13c-like_Zebrafish | VAL85, MET86, SER89, ARG92, TRP98, LEU106, HIS108, THR109, ASP112, LEU113, PHE114, THR116, VAL167, VAL168, LYS171, ALA172, ASN173, GLU175, LEU177, GLU178, GLU179, TYR180, ILE181, ALA182, GLY188, GLY189, CYS190, THR191, LEU192, TRP198, SER199, ASP202, PHE272, PHE291, ASP292, PHE294, GLY295, TRP296 |

**Table S3. Zebrafish binding contact residues.** 38 contact residues were identified in the upper third portion of the transmembrane domain of TAAR13c using CB-dock2 (Liu et al., 2022).

| **Species** | **Receptor** | **I-TAASER C-score** |
| --- | --- | --- |
| Cloudy catshark | OR1 | -0.68 |
|  | ORA4 | 0.03 |
|  | TAAR1a | -0.71 |
|  | V2R1 | -0.80 |
|  | V2R19 | n/a |
|  | V2R25 | -0.61 |
|  | V2R34 | -0.52 |
| White shark | OR1 | -0.88 |
|  | ORA4 | 0.12 |
|  | TAAR1a | 0.08 |
|  | V2R1 | -0.37 |
|  | V2R19 | 0.10 |
|  | V2R25 | 0.56 |
|  | V2R34 | -0.01 |
| Zebrafish | TAAR13c-like | -0.86 |

**Table S4. Olfactory receptor 3D models with confidence scores (C-score).** 3D model for each receptor was predicted using I-TASSER and selected for protein docking, I-TASSER predicts up to 5 models per receptor, the top scoring model for each receptor is chosen for docking analysis.

| **Receptor** | **Species** | **Ligand binding site** |
| --- | --- | --- |
| OR1 | Cloudy catshark | 83:A, 99:A, 103:A, 104:A, 107:A, 108:A, 196:A, 200:A, 204:A, 250:A, 253:A, 254:A, 257:A, 276:A, 277:A, 280:A |
|  | White shark | 80:A, 100:A, 103:A, 104:A, 107:A, 108:A, 178:A, 200:A, 204:A, 250:A, 254:A, 257:A, 276:A, 280:A |
| ORA4 | Cloudy catshark | 83:A, 84:A, 87:A, 88:A, 184:A, 185:A, 188:A, 192:A, 249:A, 252:A, 253:A, 256:A, 275:A, 279:A |
|  | White shark | 64:A, 67:A, 84:A, 87:A, 88:A, 91:A, 92:A, 171:A, 188:A, 192:A, 249:A, 253:A, 256:A |
| TAAR1a | Cloudy catshark | 113:A, 114:A, 117:A, 118:A, 165:A, 195:A, 201:A, 204:A, 205:A, 208:A, 209:A, 274:A, 277:A, 278:A, 300:A, 303:A, 304:A |
|  | White shark | 108:A, 112:A, 113:A, 116:A, 191:A, 193:A, 200:A, 203:A, 207:A, 276:A, 277:A, 280:A, 295:A, 296:A, 299:A, 303:A |
| V2R1 | Cloudy catshark | 150:A, 151:A, 152:A, 173:A, 174:A, 175:A, 223:A, 302:A, 303:A, 423:A |
|  | White shark | 80:A, 150:A, 151:A, 152:A, 173:A, 174:A, 175:A, 223:A, 302:A, 303:A, 423:A |
| V2R19 | Cloudy catshark | Non-functional |
|  | White shark | 148:A, 149:A, 150:A, 171:A, 172:A, 173:A, 221:A, 300:A, 301:A, 402:A |
| V2R25 | Cloudy catshark | 83:A, 87:A, 157:A, 158:A, 159:A, 180:A, 181:A, 182:A, 230:A, 309:A, 408:A |
|  | White shark | 67:A, 68:A,69:A, 105:A, 106:A, 107:A, 155:A, 209:A, 234:A, 235:A, 337:A |
| V2R34 | Cloudy catshark | 78:A, 82:A, 152:A, 153:A, 154:A, 175:A, 176:A, 177:A, 225:A, 304:A, 405:A |
|  | White shark | 69:A, 139:A, 140:A, 141:A, 162:A, 163:A, 164:A, 212:A, 291:A, 292:A, 392:A |
| TAAR13C | Zebrafish | 81:A, 85:A, 89:A, 98:A, 108:A, 109:A, 112:A, 113:A, 116:A, 199:A, 202:A, 269:A, 272:A, 276:A, 295:A, 298:A, 299:A |

**Table S5. Olfactory receptor predicted ligand binding sites.** The predicted ligand binding sites were predicted by COFACTOR (Roy et al., 2012; Zhang et al., 2017) and COACH (Yang et al., 2013).

| **Species** | **Receptor** | **Blood-decenal** | **Cadaverine** | **Melanin** | **Pavoninin_4** | **Putrescine** | **Taurine** |
| --- | --- | --- | --- | --- | --- | --- | --- |
| Cloudy catshark | OR1 | -5.6 | -3.9 | -9.4 | -7.8 | -3.5 | -3.8 |
| Cloudy catshark | ORA4 | -5.1 | -4.2 | -6.9 | -7 | -3.8 | -4.4 |
| Cloudy catshark | TAAR1a | -5.2 | -4.4 | -8.3 | -10 | -3.8 | -3.9 |
| Cloudy catshark | V2R1 | -6.4 | -3.9 | -10.6 | -7.7 | -3.4 | -3.9 |
| Cloudy catshark | V2R19 | n/a | n/a | n/a | n/a | n/a | n/a |
| Cloudy catshark | V2R25 | -5.4 | -3.7 | -9.9 | -6.7 | -3.4 | -3.9 |
| Cloudy catshark | V2R34 | -5.7 | -4 | -8.7 | -8.6 | -3.6 | -4.1 |
| White shark | OR1 | -6 | -3.9 | -7.4 | 3.9 | -3.6 | -4.3 |
| White shark | ORA4 | -5.4 | -3.8 | -9.2 | -7.6 | -3.3 | -3.8 |
| White shark | TAAR1a | -6.1 | -3.5 | -7.3 | -2.9 | -3.4 | -3.7 |
| White shark | V2R1 | -5.2 | -3.8 | -11 | -9.9 | -3.7 | -4.5 |
| White shark | V2R19 | -6 | -4.2 | -9.7 | -8.6 | -3.7 | -4 |
| White shark | V2R25 | -5.9 | -3.8 | -9.2 | -8.4 | -3.5 | -4.5 |
| White shark | V2R34 | -5.4 | -3.9 | -9.3 | -9.6 | -3.5 | -4.4 |

**Table S6. Molecular docking analysis.** Binding affinity scores between each receptor-odorant combination. The cloudy catshark contains a non-functional (n/a) receptor, V2R19.

**Supplementary Figure**s


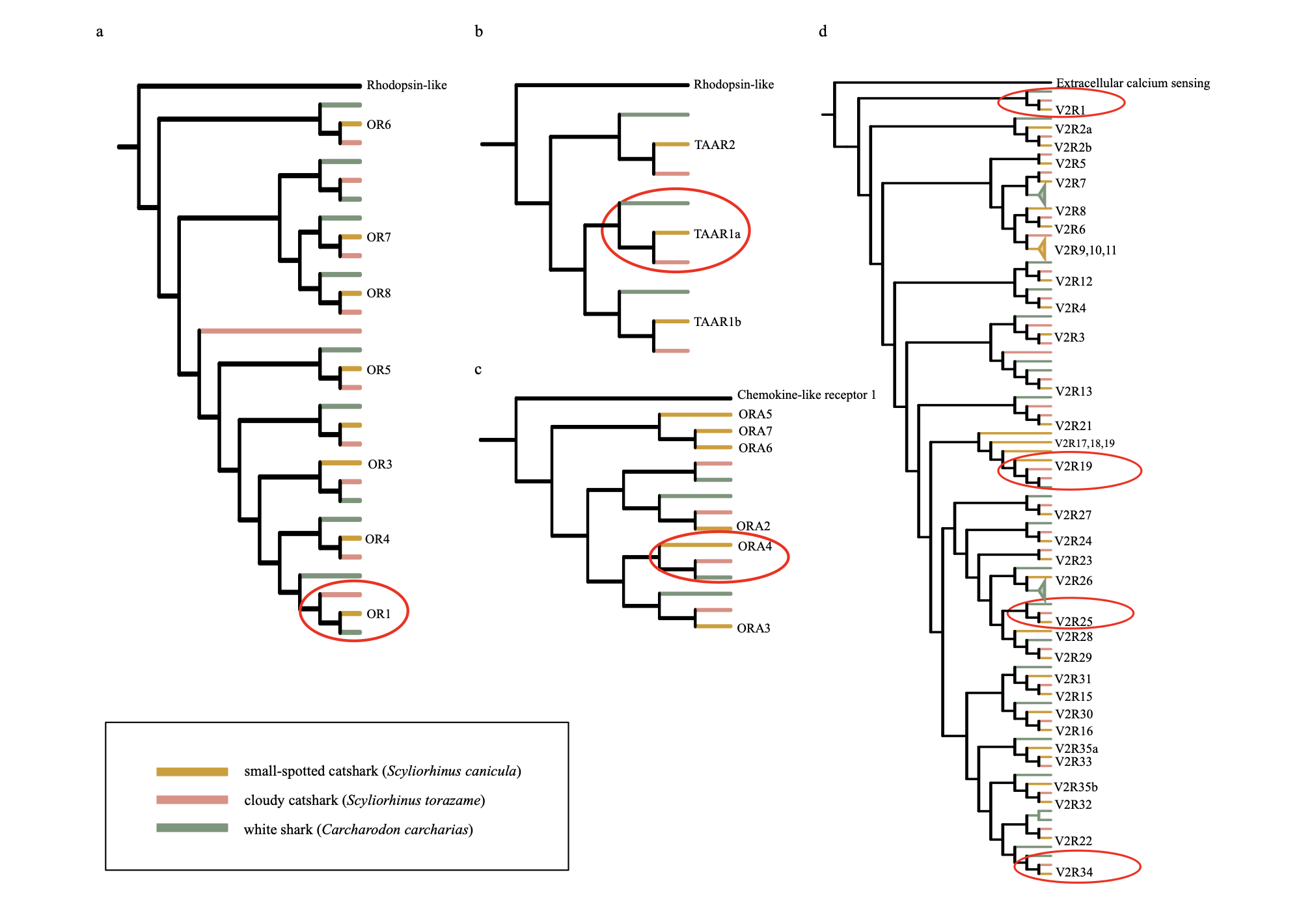


**Figure S1. Phylogenetic position of olfactory receptors chosen for 3D protein modelling**. Chosen receptors are indicated by a red circle positioned on the tree. The small-spotted catshark was used as a reference and sequences were taken from Sharma et al. 2019 and Syed et al. 2023. Each phylogenetic tree was computed with IQ-TREE with 1000 ultrafast bootstraps and the best model. **a)** ORs were rooted using a Rhodopsin-like receptor from *Branchiostoma lanceolatum* (ref_XP_066271873.1). **b)** TAARs were rooted using a Rhodopsin-like receptor from *Branchiostoma lanceolatum* (ref_XP_066278000.1). **c)** ORAs were rooted using a chemokine-like receptor 1 from *Branchiostoma floridae* (ref_XP_035668487.1). **d)** V2Rs were rooted using an extracellular calcium-sensing receptor-like *Branchiostoma floridae* (ref_XP_035685743.1). To infer gene trees for these receptors, the most suitable models of sequence evolution were as follows; for the ORs, the JTT model with frequency and gamma distribution (JTT+F+G4) was the best fit. For the ORAs, the mitochondrial inverted model, with frequency and gamma distribution (mtInv+F+G4), was selected. For both TAARs and V2Rs, the JTT+G4 model was utilized.

**Figure S2. Zebrafish TAAR13c docked with cadaverine.** TAAR13c structure (red) with cadaverine (yellow, two amine end groups blue (nitrogen) and white (hydrogen)). Two major interacting sites of TAAR13c with the cadaverine molecule are highlighted in cyan: Asp112 and Trp296. These two carbon chains are important for binding in the zebrafish TAAR13c protein.

**Amino Acid sequences for olfactory receptors used in 3D modelling**

**>Cloudy_catshark_OR1**

MNDSALSHTEFLLQGVPNGDGYELLIFISFLLVYVVIIAANLTIMYLVKTETKLHGPMYYLLCLLAGIDIILSNVTIPKILEMYSLQSKVISLEACVTQMFLVYCTALSESTLLVAMAYDRYVAICQPFNYHKLKLHYVLLVVALIIFLRAMCFVAVATLLTQATYCGSNFIQNCYCNYGSLSKLACDGVTVSDAITYPLSFLITLPDSSLIIISYFKIFKVAFHTGQGEARTKALNTCTSHCCVLILFYTSALFEFTMYLIPSIYSPELHFVVAVTFAIFQPIFNPIIYGVRTKEIRKSFLKLLGRGRVADRS

**>Cloudy_catshark_V1R/ORA4**

MAEHPVQVFIYALIVSCGIFGNALVLWIAADSTRENRYLASSDLILMNIAAANLMISLTRNTLLLTFDAGQSVSFSDVGCRLMMFIWTWLRSASIWVTLSLSLYHFITIRISRTMLGKLSERRKVVMALVVEWVLSLVYASFALPYSSNSKNNSTNNFMVISSTLRPLLGCVWTFPNEVSGLTYAMVSVVIHEAIPISLMVFSNSATLLFLYKHHRKTRDVQFSSSHGNTEWKAAKTILYLILLFIFSWGTHVISVNYYNFKGSPSTRYMLIIARFSASGFVGFYPLVVVSGHSKLRKKMRNILKCGWLNFVQQK

**>Cloudy_catshark_TAAR1a**

MNISLRNTETVEFCYEFVDGTSCVKATRSHGVRAALYAFGAVAILVTIFGNMLVIISISHFKQLHTPTNYLILSLAIADFLLGCMVMPYSLMRSIENCWYLGKLFCKLQASFDFMLCAASIFHLCFISVDRYYAVCDPLKYKTRITLQIVLIMIFISWILSAFVGFGMICLELNLIEIRDFYYNNIYCYGGCILVMGKVCSVIYSLISFYFPGFIMLCIYTKIYLVATKQARAIHDITRQIQAIKDSKTVTSQTSERKAAKTLGIVVGVFLICWSPYFTCNFIDPFIEHSTPPIMFDLFFWLGYLNSAFNPVIYAFFYSWFRKALKIVLTFKIFSNDSSRINLF

**>Cloudy_catshark_V2R1**

MELLNYFRFLVYFTISVAVSGKSTCKLKGTFNLNSFKTPGDVIIGGMFPIHYRVVASNSSSSTSPQSSGCEGFNFRAFRWARTMIHAINEINQNNSILPDIQLGYTIYDSCFTISKAVEGTLTYLTGQDEAVPNYRCGNGAPLAVLIGAGGSALSIATARILGLYYFPQVDYSASCSVLSDKFQFPSFIRTIPSDVFQSKAMAKLVVHFGWTWVGTIASDDDYGKYGIKNFKEEVEKVGVCISFSETIPKVYAREKIDRIVDTIQQSTAKIIAVFSADIDLSYLMEEVLRRNISGRTWIASEAWINSALISKPEYSSLLGGTIGLAIQRADIRGLQNHLIQLDPINAGEKLITEFWERAFDCMWPESGVAVSSMFDAEKTDTNRTGIKNRIHNIPPLSQRFCSGKENLYGIYNTYTDVSQLRLTYSVYKSVYTVANALHNLHTCKKGEGPFAGGSCANITNFQPWQLMYYLKNVRFRSLLGEEIYFDVNGDIDAMYDIMNWQRTSDGHISFKVVGSYNGTAPPGQEMTIQNDSILWNADQVTPPFSVCSESCQPGTRKGIRQGEPVCCFDCIPCADGEITNETDSRECIQCPEDYWSNENRDECVHKLIEYLGYNDALGMALIALSVFGACIAIAIAVAYMVRRDTPLVKANDRGLSFVLLFSLVICFLSSIVFVGLPVAWSCMTRQVLLAISFSTCLSCMLSKAVNLMLKARANKAKSPEGTEKKPLSPFQQRMITLVFVLCHACLCAAWLLILPPHPIKNTQSQNIKIIMECNEGSVIFLCCVLGYDLLLAAICFVFAFIARKLPDNFNEAKFMTFALLVFFIVWISFIPAYLSTRGKYMVAVEMFAILASSFGLLACLFVPKCYIILLKPERNTEELVKGKNDTNDKSAPPTSQSVTSSAISTTCSTVTLN

**>Cloudy_catshark_V2R25**

MRRRMLVMYPQLFWALSLFRFFPAHATDEASCERWGADDLLNLSKDGDIILGGIFRVHAEVNHRDLTFKIQPQSLTCTKFWFRFFRFALTMIFAIDEINQDSTLLPDVTLGYRIYDACTTPSLSLKAAFEFLNGRQDMSQNNPCKRTPLISAVVGDSGSSQSLAIATLIGIFRIPMVSYLSTCECLSNRKKYPSFFRTIPSDYFQAKALAQLVKHFAWTWIGTVRSDDDYGNFGMQAFTEAVQQLGVCIAFSETFHAIYSRDKLRKTIATIKNSSTKVVLAFLAQSDMEVLIKEIIRQNVTGIQWIGSEAWAATLVVPAEESKRFLSGTIGIAIRKVDIRGLKQFLMQVHPSLYPGNLLVKEFWETTFGCIFNNRGNKTSDPGIHECTGREDLQTVHNSLTDVSQYRVEYNVYKATYAIAHALHNMLSCKSRTELFSNNSCTNDLNFEPWQLLHHMKTLNFITKMGEKVNFDENGDPVPTYDIINWQTNALGETEIVDVGHYDGSAPAGQEFLINEEAMVWSGGQIMAPKAVCSESCTPGKRKATRNGEPKCCFDCLPCPEGEISNTTDATNCIKCPLEYRSNQERDRCVLKEIEFLSFGETMGIILLAVALFGACITMGVFSTFYFYKDTPVVKANNSELSFLLLVALTLCFLCSIAFIGEPSVWSCILRHTAFAVIFVLCISCVLSKTVVVVMAFNAKFPNHNMMKWFGPTQQRLTVAFLTMVQCLICTVWLSTLPPYPRKNSAYAKNRIIFECNVGSAINFYCALGYIGVLSCVCFLAAFLARQLPNNFNEAKHITFSMLIFCAVWVTFIPAYISSPGKYAVAVEVFAILASSFGLLICIFAPKCYIILFKQSENTKRHIMGH

>**Cloudy_catshark_V2R34**

MLRRSERYPLKSIVMHHISLGFLFAVAGAAANKPDCQLQGNLDMPGISENGDVILGGMFHVHSHRLERIDRFEQSPEQPLCKGQTMIFAIEEINRSSTLLPNITIGYKILDDCASSTTATQAALTLANGREELISANDCKGLPNVLAIIGGGGSSQSIAISRTIGPFGMPLISYFSTCACLSNRQEYPAFYRTIPSDYYQSKLLAQLVKRFGWTWIGTVRSNNDYGHFGMEGFLEDIEELGICVAFSVSFSRTDPREKVAEIVQVIKKSTTKVVVVFSAKREMRILLREIVRQNVSGIQWIGSEAWVTAELLTPEESTKFLTGTIGPAIHMAEVIGLREFLLQVHPSATPDNNFVTEFWETTFKCTLLSDNGFESDAAADNPQCTGRESLHGVNNAYSDISMDGSSYNVYKAVYVFAHAVHDMLVCKDGKGPFTNGTCAHISTYEPWQLLHYMQAVNFTTTSGETVYFESSGDPVAIYDLMNWQVNTKGFPEIVNVGYYDASASSGQAIVLNEEGIVWNGGKNKVPRAICSESCLPGTRKVSRKGYPVCCFDCTQCPAGEISNITDALDCIKCPLEYWSNPNKDKCFPKEIEFLSFEETLGIVLVALALGGICGTLAIAGVFLQYKETPIVKANNSELSFLLLFALTLCFLCSLTFIGQPSFWSCMLQRVSFGITFVLCISCVLTKTILVLMAFTATLPNNNLMRWFGPTQQRFGVFGLTFIQGFICTIWLSIAPPFPMKNTSYYREIIILECHVGSTMAFYCVSGYIALLSCVCFVLAFLARKLPDNFNEAQCITFSMLIFCVVWIAFIPAYVSSPGKYTVAVEVFAILASSFALLLCIFVPKCTIILLKPETNTRKHVMSKLPS

**>White_shark_OR1**

MNGSGLSHTEFILQGVPNGDGYKLLIFLSFLLLYIIIFAANLTIMFLIKTKTNLHGPMYYLLCILAGIDIILSNVTIPKILAMYSFQSKVISLEACVTQMFFVYCIALSESTLLVAMAYDRYVAICQPFNYHKLKFHYILLAVALIIFLRAMGFVAVAALLTQATYCGSNFIQNCYCNYGSLSKLACHGVTVSDAITYPLSFLITLPDSSLIIVSYFKIFKVAFYTGQGEVRNKALNTCTSHCCVLILFYTSALFEFTMYLVPSIFSSELHFVVAVTFAIFQPIFNPIIYGVRTKEIRNSFLKLLGRKRVADRL

**>White_shark_V1R/ORA4**

MAGHPVQVFVYGLLVSCGIFGNALVIWIAADSTRENHNLASSNLILMNIAMANLMLSLTRNILLLTFDAGQTVSFSDVGCRLMMFIWTWLRSASIWVTLSLRLYHFVTIRTSRSTMESLSERRKVVKTLVVEWALSLVYASFALPYSSSSKNNSTNNLMVISSTIRPLLGCVWTFPNEISGLAYTLVSVVIHEAIPVSLMIFANAATLLFLYRHYRKTQDVQFSSGHVNTEWKAAKTILYLILLFIFSWGTHVFSVNYYNFKGSPRHTTC

**>White_shark_TAAR1a**

MNASLRNTEMVEYCYDFVDGSCLKATRSNGLRMALYAFGAVAILVTMFGNLLVIISISHFKQLHTPTNYLVLSLAIADFLLGCMVMPYSLVRSIENCWYLGDLFCKLQASFDFMLCAASIFHLCFISVDRYYAVCDPLKYKTRITLQIVLIMIIISWILSAFVGFGMICLELNLIEIRDFYYDNIYCYGGCILVMGKLCSVIYSLISFYFPGFIMLCIYTKIYLVATKQARAINDITRQIQSIKESKNTASQTSERKAAKTLGIVMGVFLMCWSPYFTCNFIDPFIEHATPPIMFDLFFWLGYLNSAFNPVIYAFFYSWFRKALKMILTFKIFTTDSSRINLF

**> White_shark_V2R1**

MEPRNCLRFLICLTISVAVYGKSTCKLKGKFNLNSFKMPGDVIVGGMFPIHYRVVSSNSSSSTSPQSSGCEGFNFRAFRWARTMIHAINEINQNDSILPDILLGYTIYDSCFTISKAVEGTLTYLTGQDEAVPNYRCGSGAPLAVLIGSGGSALSIATARILGLYYFPQVDYSASCSVLSDKFQFPSFIRTIPSDIFQSRAMAKLVVHFGWTWVGTIASDDDYGKYGIKNFKEEVEKVGVCISFSETIPKVYAREKIVRIVDTIEQSTAQIIAVFSADIDLSYLMEEVMQRNISGRTWIASEAWINSALISKPEYSSLLGGTIGLAIQRADIRGLHNHLIQLDPRNSGEKLITEFWERAFDCMWPEHGVAVSRILNVKTAEYNRTDVKNRIHNIPPLSQRFCTGKEDFDEIDNTYTDISQLRLTYSVYKSVYTVANALHNMHTCKTGEGPFVNGSCANITNFQPWQLMYYLKNVRFKSLLGEEIYFDVNGDIDAMYDIMNWQRTSDGYISFKVIGSYNGTAPPGQEMTIHNDSIIWNDDQVTPPFSVCSKSCQPGTRKGIRQGEPVCCFDCILCADGEITNETDSRECIQCPEDYWSNENRDECVHKVIEYLGYNDALGMTLIALSIFGACTAAAIAVVYMVRKNTALVKANNRGLSFMLLFSLVICFLSAIIFVGLPVAWSCMTRQVLLAVSFATCLSCMLSKAVNLMLKAKATKAKSPEGTETKLLGPFQQRIIALILVLCHACLCTAWLLILPPYPIKNTQSQNIKIIMECNEGSVVFLCCVLGYDALLAAICFVFAFIARKLPDNFNEAKFMTFALLVFFIVWISFIPAYLSTRGKYMVAVEMFAILASSFGLLACLFVPKCYIILLKPERNTEELVKGKTVTNDKSAPPTSQSLTNSAISTACSTITLS

**>White_shark_V2R19**

MYLLCWVLLVLVTCMFAKDYPICRLQGRFNLPELAQDGDIIIGGIFPIHYRGELPRTSYQTQPETPRCIDFNLRAFRWAQLMVFAIEEINNDPTLLPNITLGYKVYDSCATPALALRAALTILNGQEENMTLLRCNGGSSVHALIADAGSTQSIAIARTTGLFEIPMISYFSSCMCLSNKKEFPTFFRTVPSDYFQVTAFVQLVKHFGWTWLASFGSDDDYGRLGASAFVEQVTKIGACVAFSEFLPKVNDREKILHQVELIKKSNVKVILVFAPEIDLNFLVQELVRQNVTGLQWLASEGWSTAALLSAAANSKTMGGTLGWAIRRVDIPGIKQFLVRLHPSKYPGNEYIKQFWEAVFQCTWTSYNSTGETWFGPWKHECTGQEDLKDVHNAFTDESQLRVSYNTYRAVYAVANSIHNMLQCKKGQGPFVNKTCPDISKIKPWQVLHYLKRVKFTTAFGDEVRFDVNGDPLAAYDLLNWQQAPDGNIKYVKVGQYDASSSPGNQLLIEKESIMWSGGQKMVITAKCSETCLPGTRKGARLGQPICCYDCIPCAEGEISNQTDSVDCLACHSDYWPNLKRDKCILKKIEFLSFGDTMGIILTTLSLFGACLSMAVTAVFYHYKNTPIVKANNSELSFLLLFSLVLCFLCSLLFMGQPTIRSCMLRHTAFAIIFVLCISCILGKTIMVLMAFNARLPNNNVVKWFGPLQRRLSIFFLPAIQVVICILWLNLSPPYPAKNIEYQSAIIILECNVGSTTAFYSVLGYIGLLSVMCFVLAFLARELPDNFNEAKFITFSMLIFCAVWITFIPVYINSGKYIVAVEIFAILSSSFGLLTCIFAPKVYIILLRPELNTKKNLMGRPPSNRS

**>White_shark_V2R25**

MIFAIDEINQDSTLLPNITLGYRIYDACTTPSLSLKAAFEFLNGRPGISLNNPCKRTPMVSAVVGDSGSSQSLAIATLIGIFRIPMPWIKWINTFRLTLPQVSYLSTCECLSNKKEYPSFFRTIPSDYFQARALAKLVKHFGWTWIGTIRSDDDYGNFGMQAFIEAVQQLGICIAFSETFHAIHSRDKLLKTIATIKKSSTKVVLAFLAQSDMEILIKEIIRENMTGIQWIGSEAWAATLVVPAEDSKRFLSGTIGIAIHKVDIRGLKEFFMQVHPSLYPGNLLVKEFWETTFSCIFKNRGSKTDDTKSDRWINECTGTEDLQTVHNSFTDVSQYRIEYNVYKATYAIAHALHNMLLCKNGAEQFPNNSCINDLNFEPWQLLHHMKTLSFITKMGEKVNFDENGDPVPTYDIVNWQTNSRGEVEIVDVGHYNGSAPEGEEFLINEEAMVWSGGQIVVPKATCSESCTPGKRKATRKGQPVCCFDCLPCPDGEISNTTDATSCINCPLEYRSNQKRDRCIPKEIEFLSFGETMGIILVVVALLGACITLGIFSTFYMYKDTPVVKANNSELSFLLLVALMLCFLCSITFIGEPSVWSCILRHTAFAVIFVLCISCVLSKTVVVVMAFNAMHPNHNIMKWFGPTQQRLTVILLTMVQCLICTVWLSTSPPYPRKSSDYAKDIITFECNVGSVINFYCALGYIGFLSCVCFVVAFLARQLPNNFNEAKHITFSMLIFCAVWITFIPAYISSPGKYAVAVEVFAILASSFGLLLCIFAPKCYIIMFKQAENTKRHIMGNKGSQTL

**>White_shark_V2R34**

MMHPFLLWYIFTASVTATNQTTCQLQGNFNLAGISKAGDITLGGMFNVHLHRFETSRHLEQSPEQTRCKGLTMIFAIEEINRSRTLLSNITIGYKILDDCSSPTIATKAALTLANGMEEIISASDCNGLPNVLAIVGGGESSQSIAIARTIGPFRMPLISYFSTCACLSNRQEYPAFYRTIPSDYYQSKLLAQLVKTFGWTWIGAVRSNNDYGNFGMEAFMEDIEKLGICLAFSESFSRTDPQEKVTEIVQVINKSTTKVIVAFSATGEMRILLREIVRQNVTGIQWIGSEAWVTAELLSPGESTKFLTGTIGPAVHVAEVLGLRDFLLQVHPSTYPGNSLVAEFWETTFKCTLLSGNKVKSDVVPENPQCTGHESLHGVNNAYSDITMDGSSSNVYKAVYAFAHAVQEMLACEDGKGPFTNGTCAHFSTYEPWQLLHYMQSVNFTTSSGDKIYFDSNGDPVAMYDLINWQVNNHGYPVLVTVGYYDASVTSGQSITLNKEAVVWNGGNNKVPRAVCSENCPPGTRKVSRKGQPICCFDCTQCADGEFSNTTDALDCIQCPLEYWPSTNKDKCVAKEIEFLDFGETLGIVLVTLALVGICGTIAIAGVFLQHKDTPIVKANNSELSFLLLFALTLCFLCSLTFIGEPSFWSCVLRRVIFSITFVICISCILAKTILVLMAFKATLPNNNLMRWFGPTQQRFGVVGLTFIQGFICTVWLSIAAPFPMKNTSYYNEIIILECRVGSITAFYCVSGYIAILSCVCFVLAFLARKLPNNFNEAQCITFSMLIFCAVWITFIPAYLSSPGKYTVATEVFAILASSFALLLCIFVPKCIIILFKPENNTRKHVMSKMASKNL

## Supplementary References

Liu Y, Yang X, Gan J, Chen S, Xiao Z-X, Cao Y. 2022. CB-Dock2: improved protein–ligand blind docking by integrating cavity detection, docking and homologous template fitting. Nucleic Acids Research. 50. doi:https://doi.org/10.1093/nar/gkac394.

Roy A, Yang J, Zhang Y. 2012. COFACTOR: an accurate comparative algorithm for structure-based protein function annotation. Nucleic Acids Research. 40(W1):W471–W477. doi:https://doi.org/10.1093/nar/gks372.

Sharma K, Syed AS, Ferrando S, Mazan S, Korsching SI. 2019. The Chemosensory Receptor Repertoire of a True Shark Is Dominated by a Single Olfactory Receptor Family. Embley M, editor. Genome Biology and Evolution. 11(2):398–405. doi:https://doi.org/10.1093/gbe/evz002.

Syed AS, Sharma K, Policarpo M, Ferrando S, Casane D, Korsching SI. 2023. Ancient and Nonuniform Loss of Olfactory Receptor Expression Renders the Shark Nose a De Facto Vomeronasal Organ. Molecular Biology and Evolution. 40(4):msad076. doi:https://doi.org/10.1093/molbev/msad076.

Yang J, Roy A, Zhang Y. 2013. Protein–ligand binding site recognition using complementary binding-specific substructure comparison and sequence profile alignment. Bioinformatics. 29(20):2588–2595. doi:https://doi.org/10.1093/bioinformatics/btt447.

Zhang C, Freddolino PL, Zhang Y. 2017. COFACTOR: improved protein function prediction by combining structure, sequence and protein–protein interaction information. Nucleic Acids Research. 45(W1):W291–W299. doi:https://doi.org/10.1093/nar/gkx366.
